# Responding to the accumulation of adverse childhood experiences in the wake of the COVID-19 pandemic: implications for practice

**DOI:** 10.1017/cha.2020.27

**Published:** 2020-05-18

**Authors:** India Bryce

**Affiliations:** School of Education, University of Southern Queensland, Toowoomba, Australia

**Keywords:** accumulation, cumulative harm, child maltreatment, COVID-19, pandemic, child abuse and neglect

## Abstract

In early 2020, the world as we knew it began to change dramatically and rapidly with the COVID-19 outbreak. Social distancing restrictions and lockdown measures have been the most effective course of action and an inarguably imperative approach at this time. However, in trying to keep the global population safe, social distancing measures unwittingly placed children already experiencing maltreatment and disadvantage in harm’s way. This paper will consider the evidence base which attests to the importance of considering the *accumulation* of adversity when seeking to understand risk and impact of child maltreatment and disadvantage. Given the unique and unprecedented circumstances which have accompanied the COVID-19 outbreak, and the dearth of research pertaining to the impact of pandemics on child welfare, the paper draws on an emerging body of literature about the effect of natural disasters, conflict and significant global events on child maltreatment. The paper synthesises the research to date in order to call attention to the cumulative impact of the COVID-19 pandemic on children already experiencing abuse and neglect. The paper concludes with an outline of the implications for practice in the helping professions.

## Introduction

In early 2020, the world as we knew it began to change dramatically and rapidly. As the COVID-19 pandemic gained momentum globally, many struggled to comprehend the magnitude of this global event. New language was filtering through our professional and personal dialogue, words like ‘social distancing’, ‘self isolation’ and ‘flattening the curve’ became common place in our conversations, and we, as a global society, appeared to have entered a ‘new normal’. With the COVID-19 outbreak came a new language, a new set of cultural rules and expectations, new laws to navigate and new threats to tackle. A climbing global infection rate in the millions, a death toll exceeding 100,000, border shut-downs nationally and internationally, supply shortages, stock market crashes and talk of impending global economic collapse. Life felt very much like an apocalyptic disaster movie.

However, as the Australian community began to settle into this new COVID-19 world, a new disquieting concern was emerging, permeating the human services, helping professions and the front-line workforce who knew Australia’s most vulnerable intimately… *what about those already at risk, what about the already vulnerable?*

The social distancing policies and lockdown measures that were implemented to reduce the spread of infection have proven to be the most effective response. Research has proven social distancing initiatives and policies in response to the COVID-19 pandemic have substantial benefits, and enforced measures reduce the infection rate significantly (Greenstone & Nigam, 2020; Razzak, 2020). However, in trying to keep the global population safe, social distancing measures have unwittingly placed children already experiencing maltreatment and disadvantage in harm’s way. The National Centre for Injury Prevention and Control (NCIPC), Division of Violence Prevention (2020), lists a number of risk factors for child maltreatment, namely social isolation, family and parenting stress, concentrated community disadvantage and poor social connections, all factors affected during the COVID-19 pandemic. Vulnerable children now faced a new threat to their safety and well-being. Early evidence indicates that the conditions of isolation implemented to ‘flattened the curve’ on the spread of COVID-19 effectively restricted children’s exposure and access to vital services and sources of monitoring, removed their supports and placed at-risk children in an intense environment of exacerbated stressors and risk (Cluver et al., 2020; NSW Health, 2020).

This paper will consider the evidence base which attests to the importance of considering the *accumulation* of adversity when seeking to understand risk and impact of child maltreatment and disadvantage, especially in the context of the COVID-19 pandemic. Given the unique and unprecedented circumstances which have accompanied the COVID-19 outbreak, there is a dearth of research pertaining to the impact of pandemics on child welfare. However, the paper will draw on an emerging body of valuable and relevant literature about the effects of natural disasters, conflict and extreme global events on child maltreatment which accurately reflects the current pandemic context. The paper seeks to synthesise the research to date in order to call attention to the cumulative impact of the COVID-19 pandemic on children already experiencing abuse and neglect. The paper will conclude by outlining the implications for practice in the helping professions. Helping professions, in the context of this paper, are defined as those professions that respond to the welfare of individuals and address challenges in a person’s physical, psychological, intellectual and emotional well-being. These professions include, but are not limited to, child protection, social work, human services, psychology, counselling, health and education (Egan & Reece, 2018). Marrying together an understanding of the role that accumulation plays in childhood adversity, and the impact of the COVID-19 pandemic and associated social distancing measures on already chronic and cumulative abuse experiences, will better inform service delivery in a post-pandemic world.

## Accumulation – a condition of victimisation

Newman and Blackburn (2002) argued that children may often be able to overcome and grow from single episodes of maltreatment; however, as risk factors accumulate, a child’s capacity to endure them diminishes. Empirical research, and our lived experience as practitioners on the front line, supports the notion that an accumulation of risk and harm is far more predictive and far more valuable in informing practice, than viewing these adversities and violations in isolation (Appleyard et al., 2005; MacKenzie et al., 2011a; MacKenzie et al., 2011b). Cumulative risk is well accounted for in the literature and assumes that the accumulation of risk factors, rather than any single risk factor, has a higher predictive power for negative outcomes (Li et al., 2014; MacKenzie et al., 2011a, 2011b). Consider then, the risk factors for maltreatment outlined by the NCIPC (2020) – social isolation, family and parenting stress, concentrated community disadvantage and poor social connections – in the context of the social distancing conditions imposed throughout the pandemic, which began in March 2020 and continued for several months. A prolonged period in which risks already acknowledged as increasing the vulnerability of children are further compounded by environmental factors which enforce the very conditions which foster these specific risk factors.

The cumulative risk hypothesis argues that the greater the number of risk factors, regardless of their type or nature, the greater the prevalence of clinical and developmental issues (Rutter, 1979; Rutter et al., 1976; Sameroff, 2000; Sameroff et al., 1987). The ground-breaking Isle of Wight Study (Rutter, 1979; Rutter et al., 1976) identified six factors significantly correlated with childhood psychological disorders: severe marital conflict, low social status, large family size, paternal criminality, maternal mental illness and out of home care placement. The authors also revealed that no single factor was associated with increased risk for disorder, rather an accumulation of two factors, of any type, contributed a four-fold increase in the likelihood of mental disorder, and four or more factors presented a 10-fold increase (Rutter, 1979; Rutter et al., 1976). Complementary findings from the Rochester Longitudinal Study (RLS) (Sameroff, 2000; Sameroff et al., 1987) demonstrated multiple risk factors potentiated progressively poor outcomes. The RLS clearly illustrated the life-course implication resultant from concurrent accumulated risk factors present in preschool age children, as well as in adolescents.

Similarly, the dose-like relationship between childhood adversity and negative adult outcomes is clarified in the Adverse Childhood Experiences (ACE) Study that purports the more adversity a child experiences, the poorer the health and behavioural outcomes are likely to be across the lifespan (Edwards et al., 2003; Felitti et al., 1998). Extant literature emphasises the way that multiple risk factors increases the likelihood of deleterious impacts across a child’s lifespan, and illustrates how the COVID-19 pandemic could exacerbate the already harmful circumstances experienced by maltreated children. These mechanisms will be examined in more depth later in the article.

As risk accumulates, so too does harm endured through childhood adversity. Bromfield et al. (2007) coined the term *cumulative harm* to describe the profound and exponential effects of an accumulation of adverse experiences in a child’s life. Finkelhor, Ormond and Turner (2007a) proposed that for many children, ‘victimisation is more of a condition than an event’ (p.9).

Persistence is a pathway in which child maltreatment, domestic violence, family conflict, and disruption propel children into an intensively and generalized victimized condition that in turn generates anger and aggression, which, by fuelling and sustaining defiant, challenging, rule-violating behaviour, tends to lock them into an even more persistent victimized condition. (Finkelhor et al., 2007b, p.493)

Gilmore (2010) likened cumulative harm to global warming, in that it is a seemingly intractable problem, involving a culmination of human and environmental factors. When perpetrators have increased, unfettered access, when the already strained family unit is forced into isolation, existing tensions are intensified. When unemployment and financial stress contribute to an already volatile environment and when previously relied upon sources of monitoring, routine and safety, such as schools and childcare agencies are removed, children are at greater risk. The harm a child experiences through maltreatment may be significantly increased when the environment closes in, such as occurred during the COVID-19 pandemic.

## Applying a social ecological model to the COVID-19 pandemic

The primary goal of child maltreatment intervention is to prevent maltreatment from occurring by reducing factors that expose children to risk and enhancing those which mitigate against victimisation (National Research Council, 1993; Sistovaris et al., 2020). The social–ecological model, which has informed preventative approaches for several decades, organises this community of factors into nested systems that interact and transact to influence the experiences of the child in the world. In their analysis of the COVID-19 context and its impact on service delivery, the Alliance for Child Protection in Humanitarian Action (ACPHA, 2019) found that pandemics such as COVID-19, and measures taken to control the spread of disease, drastically alter the environment, and associated systems in which children exist. This increased their vulnerability to abuse, neglect, violence, exploitation, psychological distress and impaired development (ACPHA, 2019). According to Fischer et al. (2018), ‘disruptions to families, friendships and the wider community can have detrimental consequences for children’s well-being, development and their protection’ (p.9). Some of the restrictions that have a direct influence on the ecological systems which contribute to risk and protection included ‘shelter in place’ orders and lockdown measures which restrict the distance individuals can travel and the reasons for leaving home, restriction on the number of people who can convene outside the home and the number of visitors that are allowable inside a home (Australian Government, 2020).The ecological systems which contribute to child maltreatment are highly influential in the context of COVID-19 due to the specific impact the pandemic has on the elements which are contained within each level of individual, family, community, society and social norms.

## The trajectory of accumulation in the COVID-19 pandemic

Given the unique and unprecedented circumstances which have accompanied COVID-19, there is a paucity of research pertaining to the impact of pandemics on child welfare. However, an emerging body of valuable and relevant literature on the effect of natural disasters, conflict and extreme global events on child maltreatment speaks to the propensity for childhood adversity and maltreatment to increase during times such as these. United Nations International Strategy for Disaster Reduction (UNISDR, 2009) defines *disaster* as ‘A serious disruption of the functioning of a community or a society, leading to one or more of the following: human, material, economic and environmental losses and impacts’ (p.9). This definition includes natural disasters and humanitarian emergencies, as well as conflicts, and reflects accurately the recent pandemic context. The research in this field illustrates a trajectory of accumulation which involves increased stressors, reduced protective factors and, unique to this pandemic, a surge in online activity of children, which increases their accessibility to virtual abusers.

## Increased stressors and risk factors

The NCIPC (2020) specifies a range of familial risk factors which contribute to child maltreatment including social isolation, family stress such as separation, divorce, or violence and parenting stress, such as poor parent–child relationships and conflictual interactions. Similarly, significant contributing community factors are identified as concentrated neighbourhood disadvantage, such as high poverty, high unemployment rates and high density of alcohol outlets and poor social connections. The social restrictions placed on families to control the spread of COVID-19 have directly compounded the familial and community risk factors which are already acknowledged as contributing to an environment conducive to maltreatment. By its very nature, the pandemic and associated social changes have created an accumulation of adversity that was already present in the lives of vulnerable families, but this has been added to through an intensification of risk factors and familial stress. Experts have already called attention to the expected escalation in family violence during the COVID-19 pandemic as a result of additional household stressors identified as risk factors for family violence, such as loss of employment and family income, increased alcohol use among household members, closure of schools and extracurricular activities, and family members being forced to spend increasing amounts of time together (Fitz-Gibbon & Meyer, 2020; Meyer, 2020).

The Australian Bureau of Statistics (ABS, 2020) reported on the household impacts of the COVID-19 pandemic at the beginning of April, 2020. By the first week of April, 56 per cent of respondents were working paid hours, in comparison to 64 per cent in early March before the current restrictions came into force (ABS, 2020). These statistics also revealed that eight per cent of respondents were still employed but no longer working paid hours (ABS, 2020). It is also important to consider the increased stress of additional work, not just the loss of work, during the pandemic. The ABS (2020) reported that 12 per cent of people still in a job worked longer hours than usual in the first week of April due to the COVID-19 outbreak. These statistics speak to the increase of household stress due to financial hardship or increased work-related obligations impacting households during the pandemic. Australian Data commissioned by the Foundation for Alcohol Research and Education (FARE, 2020) highlighted that 20 per cent of households reported buying more alcohol than usual since the COVID-19 outbreak. In those households, 70 per cent report drinking more alcohol than usual since the COVID-19 outbreak, 34 per cent indicated they were now drinking alcohol daily and 28 per cent report drinking alcohol to cope with anxiety and stress. Studies have found that alcohol is frequently involved in domestic and family violence, and alcohol use is associated with a higher chance of physical violence and of injury (Curtis et al., 2019). According to the Australian lay press, google searchers on domestic violence have increased by 75 per cent since the first reported case of COVID-19, there has been an 11 per cent increase in calls to 1800RESPECT helpline and 26 per cent rise in calls to Mensline compared to the previous year (Doran, 2020).

A systematic review of child abuse in natural disasters and conflicts revealed that the increase in social and economic pressure, which are known to be risk factors for child abuse, exposed children to a higher rate of violence (Seddighi et al., 2019). Seddighi et al. (2019) also identified that a history of exposure to parental violence, parental substance misuse and poverty were predictors of increased violence against children during emergency situations. Studies have confirmed that children are more likely to experience physical violence in disasters due to an escalation of psychological pressures on family members (Catani, Jacob, et al., 2008; Catani, Schauer, et al., 2008; Curtis et al., 2000; Saile et al., 2014; Sriskandarajah et al., 2015). Families affected by disasters both socially and economically, especially those in low socioeconomic communities, commit violence against children more frequently (Biswas et al., 2010; Catani, Jacob, et al., 2008; Curtis et al., 2000; Sriskandarajah et al., 2015). Seddighi et al. (2019) emphasised the cumulative impact of the accumulation of stressors resulting from the unprecedented circumstances of extreme global events, concluding that polyvictimization, or exposure to multiple types of abuse like physical violence, neglect and maltreatment, was more common in disasters.

An example of the cumulative causal relationship between increased stressors and child maltreatment is evidenced in the *critical incident chain perspective* (Browne, 2002; Frude, 1991), in which a sequence of events might provoke a caregiver to maltreat a child. In the context of COVID-19, a stressful event might occur in the family home which is chronic (e.g. poverty or unemployment due to COVID-19 job loss) and distresses the carer; the carer feels a secondary stress as their expectations are incongruent with the experience of disadvantage and stress; anger and distress result and may lead to poor impulse control and maladaptive coping, potentially leading to maltreatment of the child. This perspective is often used to illustrate the sequential pattern of factors which can result in Abusive Head Trauma (AHT), colloquially referred to as Shaken Baby Syndrome.

AHT is the leading cause of death from child abuse and the most common cause of severe traumatic brain injury (TBI) in infants (Barlow & Minns, 2000; Duhaime, et al., 1998; Ellingson et al., 2008; Keenan et al, 2003; Keenan et al, 2004). Poverty and stress are both acknowledged as risk factors for AHT (Hillson & Kuiper, 1994; Kotch et al., 1995). Keenan et al. (2004) identified an increase in both AHT and non-inﬂicted TBI after Hurricane Floyd, in the USA. Berger et al. (2011) identified that AHT increased significantly during the economic recession in the US in 2008. This further highlights the effects of cumulative stress on violence and emphasises the likelihood of chronic child maltreatment during the COVID-19 pandemic.

Researchers have often mapped a pathway from feelings of powerlessness to acts of aggression, and Finkelhor (1983) described child abuse as ‘acts carried out by abusers to compensate for their perceived helplessness or loss of power’ (p. 19). Extreme global events, such as political conflict, disasters and pandemics disrupt the usual processes and patterns of everyday life, interrupting routines and reducing individuals feeling of autonomy and control over their day to day lives. This can often result in increased stress, helplessness and frustration (Miller & Kraus, 1994; Tobin & Ollenburger, 1996). According to Bugental et al. (1989) ‘catastrophic life events are more likely to lead to ineffective coping strategies among individuals who have a low sense of their own control’ (p. 263). Reflective again of critical incident chain perspective, children may become targets of the aggressive behaviours which result from a parent’s frustration with events out of their control, such as restrictions enforced in the COVID-19 pandemic (Greenwald et al., 1997). Bugental et al. (1989) reported that when parents are confronted with the realities of economic and social adversity, and feel helpless to control life events, they are more likely to perpetrate physical abuse.

As social and economic stressors and household adversity accumulate during the COVID-19 pandemic, the risks of maltreatment experienced by children residing in already volatile and risky home environments escalates exponentially. Cumulative harm is caused by ‘a series or pattern of harmful events and experiences…with the strong possibility of the risk factors being multiple, inter-related and co-existing over critical developmental periods’ (Miller, 2007, p.1). As stressors build, maltreatment escalates, increasing in frequency, duration and severity, key indicators of cumulative harm (Bromfield et al., 2007).

## Social distancing and the impact on protective factors

A central tenant of harm reduction is to increase the protective capacity of the familial and social networks of children. The CDC (2020) lists a number of factors known to lessen the likelihood of children being abused or neglected. Of particular note are protective factors which include supportive family environment and social networks, parental employment, access to health care and social services and caring adults outside the family who can serve as sources of modelling and monitoring for children. These are all elements which have been affected by the measures implemented in response to the COVID-19 pandemic.

The restrictions imposed to ‘flatten the curve’ have essentially reduced protective factors previously relied upon to buffer against child maltreatment. The isolation policies implemented at state and federal levels have further increased opportunities for secrecy and silence and, as isolation continues, vulnerable families are becoming increasingly invisible. Opportunities for proactive help seeking have been restricted and access to external support agencies, previously in a position to identify cues or warning signs, is no longer accessible or readily available (ACPHA, 2019). Schools, once relied upon as protective factors and sources of monitoring and safety for at-risk children, have been temporarily closed for a majority of children (ACPHA, 2019). Many critical front-line child protection agencies have resorted to virtual technology to monitor children’s protection needs, preventing workers from using the non-verbal cues, body language and environmental assessments to ascertain children’s safety and level of risk.

While extreme events often elicit an outpouring of organised helping in the form of economic bailouts, social media campaigns and government initiatives, existing social networks are disrupted and formerly relied upon professional supports are terminated (Curtis et al., 2000; Kaniasty & Norris, 1995). The social support deterioration deterrence model (Kaniasty & Norris, 2004; Norris & Kaniasty, 1996) proposes that the impact of potentially traumatic events on mental health is both direct and indirect through disruption of social networks and a decline in perceptions of support availability. This model calls attention to the role of perceived and actual support during distress. The very nature of the response to the COVID-19 pandemic was one of service reduction and physical support depletion, thus the unavailability of face to face services, the closed doors of health and social care agencies, and the reduced visibility of case workers, professional supports and practitioners may give the perception of support deterioration. While social services are in fact continuing to support the vulnerable, the changing face of this service delivery has resulted in a perception that vulnerable families are untethered and cast adrift. This perception further exacerbates the cumulative impact of familial stressors and feelings of vulnerability for at-risk children.

## Increase in virtual and online child abuse

Recent articles in the lay press have reported an increase in the risk of online child abuse as a result of the rise in children’s screen time during the COVID-19 lockdown (3AW, 2020; Global News Canada, 2020; Unicef, 2020; UN News, 2020). Howard Taylor, Executive Director of the Global Partnership to End Violence against Children, a public–private collaboration between UN agencies, governments, industry, regional bodies, civil society and others, stated:

School closures and strict containment measures mean more and more families are relying on technology and digital solutions to keep children learning, entertained and connected to the outside world, but not all children have the necessary knowledge, skills and resources to keep themselves safe online (UN News, 2020, p.1).

Students globally are undertaking a hybrid version of home schooling, utilising virtual platforms to participate in lessons and classes. With the rise in time children are spending online, their accessibility to perpetrators is increased. According to Quadara et al. (2015):

Online communication facilitates contact with a large number of children, allows for the initiation and continuation of grooming, allows the perpetrator to detach from the behaviour in which they are partaking; and helps them to remain anonymous in a way that is not otherwise possible. (p. 16)

Australia’s eSafety Commission (2020) reported a spike in reports of inappropriate online behaviour since the beginning of the COVID-19 restrictions as both children and perpetrators were spending more time at home and online.

While this is a risk factor for all children, speaking cumulatively, children who have experienced childhood adversity and intra-familial abuse, and are inadequately supervised due to parental neglect, substance misuse or mental illness, are at higher risk of falling prey to extra-familial offenders (Finkehor et al, 2007a. 2007b). Given the dynamic nature of the internet and associated telecommunications technologies relied upon during the COVID-19 pandemic, consideration of the accumulation of risk factors which increases a child’s vulnerability to online victimisation is a paramount concern.

## Embedding accumulation in practice post pandemic

Extreme events, including the COVID-19 pandemic, have an immediate and prolonged impact on the organisation, delivery and all other aspects of practice in the affected area. In 2020, our affected area is global, and there is no community which has not experienced the pervasiveness of this event. Confirmed in research, extreme events can lead to increases in domestic violence (Reese, 2004; Smith, 2012), child sexual assault (Smith, 2012) and child abuse (Curtis et al., 2000) – adversities to which human services are normally intended to respond. Maglajlic (2019) highlights that responsiveness and flexibility are key considerations in a social services response to such events. Helping professionals must absorb and address new needs, as well as to meet existing ones. During and after the COVID-19 pandemic, helping professionals must also respond to the escalation and accumulation of existing adversity.

Responding to the cumulative risk and harm experienced by children during the COVID-19 pandemic requires the prioritisation of the interruption of harmful patterns of action and inaction which perpetuate the cycle of disadvantage and maltreatment experienced by at risk children. According to Bliss and Meehan (2008), such interruption must address core immediate needs, such as housing, transport and medical and social support. Triaging immediate needs is critical but an acknowledgement of the long-term implications of an accumulation of risk and harm needs to be embedded in practice in order to effectively respond to the needs of vulnerable children post pandemic (Bryce, 2018). Price-Robertson et al. (2013) suggest that a multiservice response better captures and responds to the complexity of cumulative harm. Equally, Scott (2014) argues that complex family needs can be beyond the capacity of one service, agency or government department, with multiservice collaboration delivering better practice. Thus, in order to effectively respond to the cumulative risk and harm perpetuated through the COVID-19 crisis, agencies tasked with the role of supervising and caring for vulnerable children and at-risk families must increase their collaborative capacity. This will expand monitoring and protective oversight and improve the likelihood of ameliorating the deleterious impacts of chronic and repeated maltreatment. Drawing on collaborative resources is supported by Sistovaris et al. (2020) in their recommendation for protecting children during pandemics. The authors propose working with communities to identify strategies for protecting vulnerable groups and carrying out activities to promote safe coping mechanisms and support affected populations.

Social support reflects an ongoing dynamic transaction among individuals, their social networks and environmental pressures (Cohen, 1992; Pierce et al., 1992; Vaux, 1988). Reflecting on the impact of perceived invisibility of support espoused in the social support deterioration deterrence model (Kaniasty & Norris, 2004; Norris & Kaniasty, 1996), increasing the visibility of supports and mobilising networks in a way that balances the perceived reduction in traditional social networks will reduce the stressors contributing to an environment conducive to cumulative risk and harm. According to Kaniasty and Norris (1993), ‘positive relations between life stress and support exemplify the mobilisation of social networks that, in turn, protect individuals from experiencing the negative effects of the stressor’.

## Anti-oppression and empowerment

In Maglajilic’s (2019) review of literature on social service delivery in extreme events, oppression was exposed as an important lens which survivors apply to their understanding of interactions with relevant agencies, including social services. Research highlights that while extreme events impact individuals in affected areas, their effects are disproportionately imposed upon ethnic minorities, low-income families and other vulnerable groups, such as women, children, the elderly and the disabled (Kulkarni et al., 2008; Manning and Kushma, 2016: Moore et al., 2004; Sherraden and Fox, 1997), particularly in the long term (Sundet and Mermelstein, 1997; Zakour, 1997). The cumulative experience of disadvantage and adversity of these vulnerable communities at a structural level contributes to the narrative of accumulation that exposes at-risk children to increased harm during the COVID-19 pandemic.

Practice within an anti-oppressive framework involves skills in assessing how wider systems have impacted on individuals or groups (families and communities) and the diversity of oppression they may be experiencing. Dominelli and Campling (2002) draw attention to the way in which oppression is rooted in everyday life and demand we actively reject it in our work. The COVID-19 outbreak and the restrictions that have accompanied the pandemic have exacerbated the oppression and disadvantage of our already under-served communities. Pittaway et al. (2007) highlight the importance of advocacy with, and on behalf of women and children in disaster situations, promoting human rights and gender equality as paramount issues in bringing about social transformation. This is especially relevant given the impact of the pandemic on working mothers due to the closure of schools and childcare (Alon et al., 2020).

In order to address the feelings of helplessness and powerlessness incurred as a result of the pandemic and associated restrictions, all practice in the COVID-19 climate, both now and post pandemic, should be underpinned by an empowerment approach. Empowerment refers to enabling individuals to gain control and mastery over their lives (Kieffer, 1984; Zimmerman, 2000). According to Turner and Maschi (2015), empowerment encourages individuals to band together as communities and takes action to improve their situations. Empowerment can be viewed as a theoretical framework which advocated for people take more control over their lives (AlMaseb & Julia, 2007). This approach to service delivery during the COVID-19 pandemic will assist in addressing the underlying emotional drivers motivating abusive behaviour. Supporting clients to feel more in control during these unprecedented times will reduce cumulative stressors through empowerment and address power imbalances constructed through the measures implemented to ‘flatten the curve’ on COVID-19.

## Concluding comments

While, globally, we are all experiencing a crisis during the COVID-19 pandemic, for practitioners, the ‘peak’ will occur as we emerge from isolation and the impact of this time becomes apparent. While social distancing and lockdown measures have been necessary and effective, achieving the right balance between infection control and mitigation of adverse psychosocioeconomic effects must be considered (Holmes et al., 2020; Prieto & Sacristán, 2003). For helping professionals, addressing the accumulation of adversity, disadvantage, exposure to domestic and family violence, and chronic maltreatment will be a priority in responding to the impacts of the COVID-19 context, post pandemic. Entering into this unchartered territory of service delivery will require an acknowledgment of the way in which risk and harm accumulate and an integration of this knowledge into all direct and indirect practice from this point forward. We can learn from the literature which has evolved from past extreme events and integrate this information into our practice in the context of post COVID-19. Now and into the future, adopting a collaborative multisystem approach to meeting the complex needs of vulnerable families, addressing oppression and disempowerment, and mitigating the augmentation of stressors due to the pandemic and the related measures of social distancing restrictions, will be a proactive means of harm reduction. As we emerge from this truly life-changing experience, there will be no service delivery which does not involve responding to accumulated risk and cumulative harm in some form, which will be COVID-19’s legacy to our profession. However, perhaps we will have a more accurate appreciation for the mechanisms of accumulation, and our practice will be better informed for having understood maltreatment from this enlightened perspective.
